# From genome to phenotype: advances, challenges and One Health perspectives in the genomic prediction of antimicrobial resistance

**DOI:** 10.3389/fcimb.2026.1924354

**Published:** 2026-10-21

**Authors:** Emílio Gomes, Ricardo J. Figueiredo, João R. Mesquita, Carina Almeida, Daniela Araújo, Nuno F. Azevedo, Joana Castro

**Affiliations:** 1 Laboratory for Process Engineering, Environment, Biotechnology and Energy (LEPABE), Associate Laboratory in Chemical Engineering (ALiCE), Faculty of Engineering, University of Porto, Rua Dr. Roberto Frias, Porto, Portugal; 2 National Institute for Agrarian and Veterinary Research (INIAV), Rua dos Lagidos, Vila do Conde, Portugal; 3 School of Medicine and Biomedical Sciences (ICBAS), University of Porto, Porto, Portugal; 4 Department of Chemistry and Biochemistry, Faculty of Sciences, Associated Laboratory for Green Chemistry (LAQV), Network of Chemistry and Technology (REQUIMTE), University of Porto, Porto, Portugal; 5 Centre of Biological Engineering (CEB), University of Minho, Braga, Portugal

**Keywords:** antimicrobial resistance (AMR), genomic prediction, genotype-phenotype gap, machine learning, One Health, silent resistome, whole-genome sequencing (WGS)

## Abstract

Whole-genome sequencing (WGS) has substantially improved the detection and surveillance of antimicrobial resistance (AMR) by enabling the rapid identification of acquired resistance genes, resistance-associated point mutations, and mobile genetic elements. However, the presence of a resistance determinant does not necessarily establish its functional contribution to the antimicrobial phenotype. This mini-review argues that a central limitation of current genotype-to-phenotype prediction is representational: multidimensional resistance architectures are frequently reduced to binary presence–absence profiles. Such representations disregard gene integrity, expression state, promoter architecture, copy number, genomic localization, regulatory topology, strain background, and within-isolate population heterogeneity. Using functionally silent determinants, pseudogenized resistance genes, inducible and dosage-dependent resistance, altered permeability and efflux, heteroresistance, and genomic-context dependence as examples, we examine how this loss of biological information can generate both false-resistant and false-susceptible predictions. We further discuss why metagenomic ARG detection cannot be directly equated with phenotypic resistance and why prediction models trained on restricted clinical populations may not generalize across species, lineages, and One Health compartments. Moving from determinant detection toward context-aware and quantitative phenotype prediction will require the selective integration of long-read sequencing, transcriptomics, copy-number analysis, population-aware sequencing, standardized phenotypic metadata, and externally validated computational models. The relevant unit of AMR prediction is therefore not the resistance gene alone, but its functional state within a specific genomic, cellular, population, and environmental context.

## 1 Introduction

Antimicrobial resistance (AMR) is a major global health challenge affecting human and veterinary medicine, food production, and environmental health. In 2021, bacterial AMR was associated with an estimated 4.71 million deaths worldwide, including 1.14 million deaths directly attributable to resistant infections (Naghavi et al., 2024). Phenotypic antimicrobial susceptibility testing (AST), including disk-diffusion and minimum inhibitory concentration (MIC) assays, remains the reference standard for determining antimicrobial susceptibility. However, it requires viable bacterial culture, typically delays results by 24–72 h, and measures antimicrobial response under standardized *in vitro* conditions that may not reproduce biofilm-associated growth, host-mediated stress, antimicrobial induction, or other context-dependent phenotypes (Ciofu et al., 2022; Elbehiry et al., 2025).

Whole-genome sequencing (WGS), metagenomics, and machine-learning approaches have expanded AMR detection by identifying acquired resistance genes, resistance-associated point mutations, and mobile genetic elements and by supporting transmission analysis and genomic surveillance (Boolchandani et al., 2019; Kim et al., 2022). Nevertheless, genomic detection is not equivalent to phenotypic resistance. A detected determinant may be transcriptionally or translationally attenuated, inducible, dosage-dependent, or structurally disrupted. Its phenotypic effect may also be modified by promoter architecture, membrane permeability, efflux-pump expression, mobile-element context, bacterial species, strain background, epistatic interactions, environmental conditions, and within-isolate population heterogeneity (Hughes and Andersson, 2017). Genotype-to-phenotype prediction is further constrained by incomplete or biased resistance databases, heterogeneous phenotypic metadata, limited representation of non-human and environmental isolates, and insufficient external validation across One Health settings (Mahfouz et al., 2020; Kim et al., 2022; Prosperi et al., 2022; Muloi et al., 2023).

Recent reviews have examined sequencing-based AMR detection, machine-learning prediction, genomic surveillance, and their implementation within One Health frameworks (Boolchandani et al., 2019; Kim et al., 2022; Djordjevic et al., 2024; Matsumura et al., 2025). This mini review therefore does not seek to provide another broad overview of genomic AMR surveillance. Instead, we argue that a central limitation of current genotype-to-phenotype prediction is representational: bacterial genomes are frequently reduced to binary resistance-determinant matrices, although resistance depends on the functional integrity, expression, dosage, genomic context, strain background, and population distribution of those determinants. Here, we define context-aware AMR prediction as genotype-to-phenotype inference that represents not only determinant presence but, when mechanistically relevant, its sequence integrity, allelic and regulatory state, dosage, genomic and mobile-element context, interacting strain background, expression state, and within-isolate population frequency. Using functionally attenuated resistance genes, structurally disrupted determinants and pseudogenes, inducible and dosage-dependent resistance, altered permeability and efflux, heteroresistance, and structural genomic interactions as examples, we examine how gene presence alone can generate both false-resistant and false-susceptible predictions. These mechanisms and their contributions to genotype–phenotype discordance are schematically illustrated in **Figure 1**.

**Figure 1:**
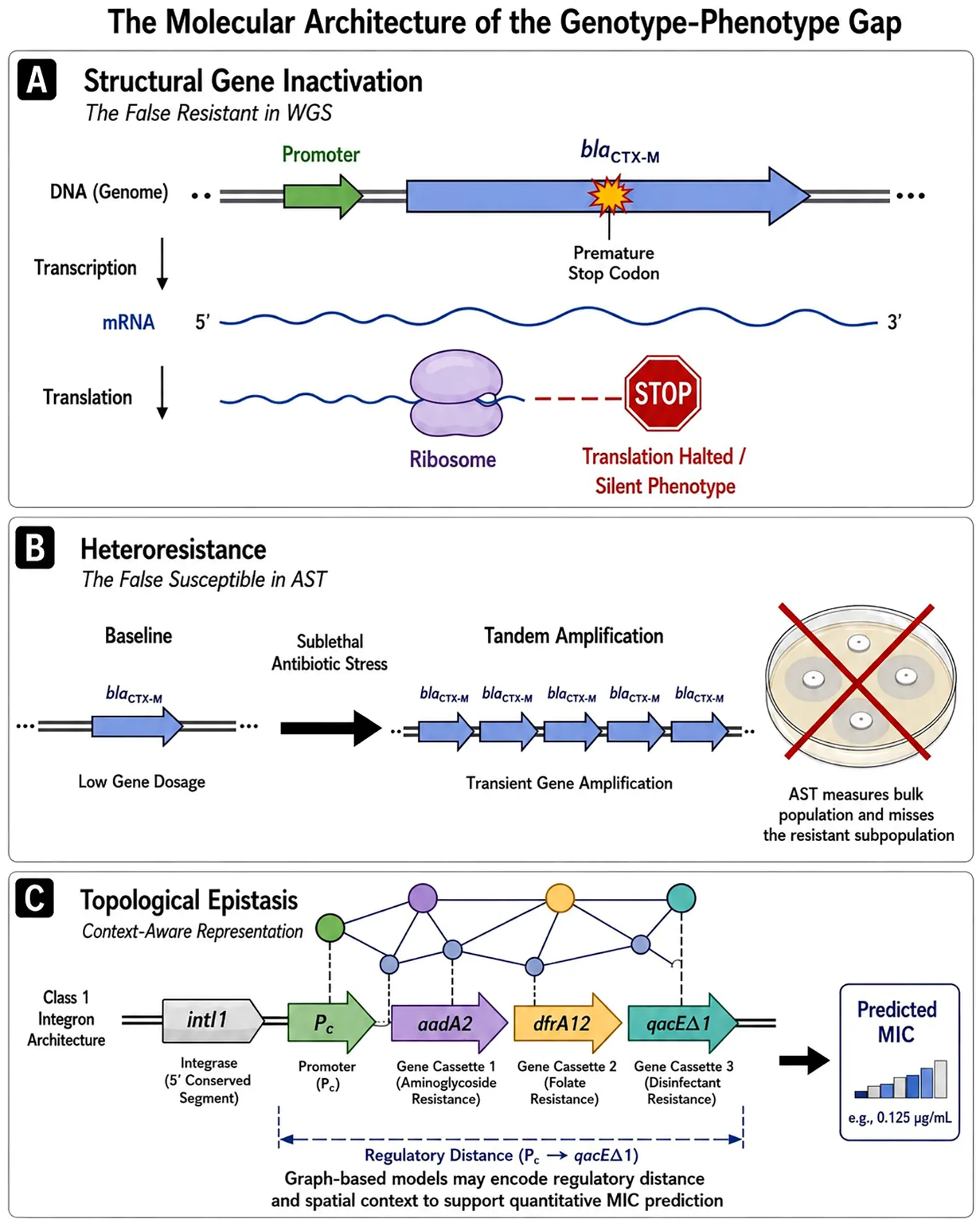
The molecular architecture of the genotype-phenotype gap. **(A)** Structural gene inactivation: a resistance determinant carrying a premature stop codon may remain detectable by whole-genome sequencing despite the absence of functional protein production, leading to a false-resistant prediction when coding-sequence integrity is not assessed. Functionally intact but attenuated determinants are discussed separately in Section 3.1. **(B)** Heteroresistance and gene dosage: gene amplification can generate minority resistant subpopulations that may be missed by routine antimicrobial susceptibility testing and consensus-genome analysis. Such amplifications may be transient and can be lost during antimicrobial-free culture or repeated passage. **(C)** Genomic topology and context dependence: gene order, promoter distance, cassette position, and interactions among resistance determinants may influence resistance magnitude. Graph-based models may encode these relationships to support quantitative MIC prediction, although their incremental value requires validation in appropriately phenotyped and independent bacterial populations.

## 2 Conditions determining the reliability of genotype-based resistance prediction

### 2.1 Sequence-based prediction and the conditions for genotype-phenotype concordance

Genotype-based prediction performs most reliably when resistance is driven by a well-characterized determinant with a large and reproducible phenotypic effect, whose presence is sufficient to generate the corresponding phenotype within a validated organism–antimicrobial context. This is particularly true for well-characterized acquired β-lactamases, including extended-spectrum β-lactamases and carbapenemases, and for established quinolone-resistance-determining region mutations in *gyrA* and *parC*, for which sequence-based tools can achieve high genotype–phenotype concordance (Boolchandani et al., 2019; Bortolaia et al., 2020).

Long-read sequencing can further strengthen these predictions by resolving complete plasmids, integrons, and chromosomal contexts that may remain fragmented in short-read assemblies, thereby improving the localization and epidemiological interpretation of resistance determinants (Wick et al., 2023; Gomes et al., 2025; van Almsick et al., 2026). These examples define the conditions under which binary gene detection is informative. However, prediction becomes substantially less reliable when resistance depends on gene integrity, expression, dosage, genomic background, population heterogeneity, or environmental induction.

### 2.2 One health as a test of model transportability

Cross-sectoral surveillance exposes genotype-to-phenotype models to distinct lineages, genomic backgrounds, and phenotypic-testing frameworks, so models developed in restricted datasets may not generalize reliably across One Health compartments (Kim et al., 2022; Muloi et al., 2023; Prosperi et al., 2022). ResFinder 4.0 achieved 97% overall concordance in a Danish animal/meat *Escherichia coli* dataset and 98% in a UK animal-surveillance dataset, whereas a blind-test dataset comprising human- and animal-origin isolates from clinical and surveillance sources yielded 95.3% overall concordance, with concordance for ertapenem falling to 54.6% (Bortolaia et al., 2020). These differences cannot be attributed to ecological compartment alone because phenotypic methods, resistance prevalence, lineage composition, and unrepresented mechanisms may also differ across datasets; nevertheless, they illustrate why predictive performance should be externally evaluated rather than assumed to transfer across settings. Likewise, a model developed using 980 pathogenic *E. coli* isolates from seven livestock and companion-animal hosts achieved a mean F1 score of 98.0%, yet fitted predictors for the same antimicrobial varied across animal hosts, indicating heterogeneity in genotype-to-phenotype relationships across host backgrounds (Chung et al., 2023). One Health surveillance should therefore be viewed not only as a sampling framework, but also as a test of the transportability and external validity of genotype-to-phenotype prediction models. **Supplementary Figure S1** summarizes the broader One Health implementation context.

## 3 Biological states hidden by binary resistome profiles

### 3.1 Functionally intact but silent or attenuated resistance determinants

A major source of genotype–phenotype discordance is the presence of structurally intact resistance determinants whose expression is absent, weak, or repressed under the tested condition. Attenuation may result from weak or absent promoters, transcriptional repression, translational attenuation, stable mRNA structures, or unfavorable codon usage (Hughes and Andersson, 2017; Postupolski et al., 2021; Deekshit and Srikumar, 2022; **Supplementary Table S1**). Here, silent or attenuated resistance denotes a structurally intact determinant with absent or low expression under the tested condition. Inducible determinants are related but mechanistically distinct because their expression can increase in response to specific stimuli and are discussed separately in Section 3.4. Neither state should be conflated with pseudogenization, in which structural disruption renders the coding sequence non-functional irrespective of expression state. Sequence-based annotation can therefore identify a genuine determinant without establishing whether it is functionally active, while restoration of a pseudogene requires genetic change rather than induction alone.

The absence of detectable expression under a single laboratory condition should not be interpreted as permanent functional inactivity. The contribution of an intact determinant may vary with antimicrobial exposure, growth phase, nutrient availability, stress responses, and host regulatory background. Genome-based prediction should therefore distinguish confirmed inactivity from unmeasured or condition-dependent activity (Hughes and Andersson, 2017; Deekshit and Srikumar, 2022).

### 3.2 Structural gene disruption and pseudogenization

Frameshifts, premature stop codons, large deletions, disruptive insertions, and functionally damaging substitutions may inactivate resistance determinants while preserving sufficient sequence similarity for homology-based detection. These sequences should be classified as disrupted alleles or pseudogenes rather than as silent or attenuated determinants (Postupolski et al., 2021; Deekshit and Srikumar, 2022). Unlike structurally intact determinants whose expression may increase after induction or regulatory change, structurally disrupted determinants are not expected to regain function through antimicrobial exposure or environmental stress alone. Functional restoration would instead require genetic events such as reversion, recombination, compensatory mutation, or acquisition of an intact allele. Their principal implication for genotype-to-phenotype prediction is therefore potential false-resistant calling when pipelines report gene presence without assessing open-reading-frame integrity, truncations, or functionally relevant sequence variation.

### 3.3 Permeability, efflux, and genomic-background dependence

Resistance magnitude also depends on intracellular antimicrobial exposure and strain background. In Gram-negative bacteria, AcrAB–TolC overexpression following *ramR* alterations and MexAB–OprM overexpression associated with *mexR* or *nalD* mutations reduce intracellular drug concentrations, whereas loss or modification of OmpK35/OmpK36 or OprD restricts influx (Ochs et al., 1999; Llanes et al., 2004; Sobel et al., 2005; Abouzeed et al., 2008; Kitchel et al., 2010; Zhang et al., 2014). These mechanisms can interact with *gyrA/parC* mutations, carbapenemases, and *blaKPC* expression or copy number, producing markedly different MICs among isolates carrying the same determinant (Bruchmann et al., 2013). WGS may identify some coding or regulatory variants, but presence–absence approaches do not directly measure efflux or porin expression; transcriptomic data may therefore improve prediction when altered expression is central (Khaledi et al., 2020). In *Klebsiella pneumoniae*, the phenotypic effect of KPC production may be amplified by loss or modification of OmpK35 and OmpK36, whereas OprD inactivation is a major contributor to imipenem resistance in *Pseudomonas aeruginosa*. Thus, the same acquired β-lactamase may be associated with markedly different MICs depending on influx, efflux, and regulatory background (Ochs et al., 1999; Kitchel et al., 2010; Zhang et al., 2014).

In Gram-positive bacteria, the contribution of NorA, NorB, NorC, and MepA similarly depends on transcriptional upregulation rather than simple gene presence (Noguchi et al., 2004). Reduced vancomycin susceptibility in *Staphylococcus aureus* may involve VraSR- or WalKR-associated cell-wall remodeling, whereas reduced daptomycin susceptibility in *Staphylococcus* and *Enterococcus* may involve *mprF*, *cls*, *dltABCD*, or *liaFSR* (Munita et al., 2012; Diaz et al., 2014; Baek et al., 2017). These polygenic phenotypes are strongly background-dependent and cannot be inferred reliably from isolated markers.

### 3.4 Inducible resistance, promoter strength, and gene dosage

Binary detection also fails when resistance depends on the timing or magnitude of expression. In the *Enterobacter cloacae* complex, *Citrobacter freundii*, *Serratia marcescens*, and related taxa, chromosomal *ampC* may remain weakly expressed until β-lactam exposure or constitutive derepression through alterations affecting *ampD*, *ampR*, or associated regulatory pathways. Likewise, erythromycin-resistant but clindamycin-susceptible *staphylococci* may harbor inducible *erm*-mediated macrolide–lincosamide–streptogramin B (MLSB) resistance that is only revealed by the D-test, whereas attenuator deletions or duplications can produce constitutive expression. Gene presence alone therefore cannot distinguish basal, inducible, derepressed, and constitutive states (Schmitz et al., 2001; Fiebelkorn et al., 2003; Flury et al., 2016; Barceló et al., 2024).

Promoter architecture and dosage further modify resistance magnitude. Tn4401 promoter variation can alter *blaKPC* transcription, while gene or plasmid copy number can increase MICs in a permeability- and background-dependent manner (Roth et al., 2011; Cheruvanky et al., 2017). *mecA*-positive *S. aureus* may also remain phenotypically susceptible until increased *mecA* expression and auxiliary mutations permit resistance (Boonsiri et al., 2020). Presence–absence profiles therefore cannot distinguish basal, inducible, derepressed, constitutive, or high-copy states.

### 3.5 Heteroresistance and population-level discordance

Heteroresistance describes a minor resistant subpopulation within an otherwise susceptible isolate and may arise through low-frequency variants, plasmid copy-number changes, or transient gene amplification (Andersson et al., 2019; Nicoloff et al., 2019). Conventional AST may miss these minority populations, producing false-susceptible results, while consensus sequencing may mask the same variants or amplifications (Xu et al., 2025).

Detection depends on the resistant-subpopulation frequency and analytical resolution. Minority variants below sampling or variant-calling thresholds may be absent from colonies selected for AST, masked in a consensus genome, or filtered as noise; transient amplifications may also disappear during antimicrobial-free culture or repeated passage (Andersson et al., 2019; Nicoloff et al., 2019). Sequencing and AST performed at different workflow stages can therefore represent different population states, and a single susceptible/resistant label may be an imperfect training target for a dynamic population.

Population analysis profiles remain among the most informative methods for confirming heteroresistance, while deep sequencing, minor-variant analysis, and normalized read depth can provide complementary evidence. However, absence of these signals in routine WGS does not exclude a resistant fraction below the validated detection limit or an unstable amplification (Kim et al., 2022; Xu et al., 2025).

There is no universal sequencing threshold for heteroresistance detection because the limit depends on sequencing depth, library preparation, variant calling, quality filters, and reporting rules. In a multicenter proficiency study of *Mycobacterium tuberculosis* mixtures, the agar-proportion method detected a 1% resistant subpopulation, whereas routine WGS workflows consistently reported rifampicin-resistance variants at 10% but not 1%; laboratories also differed in whether detected variants were included in final resistance reports (Danchuk et al., 2024). These values are organism- and workflow-specific rather than general WGS cut-offs, underscoring the need to report validated allele-frequency limits of detection and bioinformatic thresholds.

Together, silent determinants, pseudogenes, expression-dependent mechanisms, genomic background, and heteroresistance demonstrate why resistance cannot be represented adequately as independent binary features.

## 4 From binary annotation to context-aware phenotype prediction

### 4.1 Binary resistome matrices as a weak representation of resistance biology

Genotype-to-phenotype pipelines frequently encode genomes as binary matrices indicating whether predefined determinants are present or absent. Although convenient, this representation omits coding-sequence integrity, allelic identity, promoter architecture, expression, copy number, genomic localization, gene order, regulatory proximity, permeability, strain background, epistasis, and population frequency (Boolchandani et al., 2019; Mahfouz et al., 2020). Isolates with identical resistome profiles may therefore display different MICs, while phenotypically similar isolates may reach resistance through different mechanisms (Hughes and Andersson, 2017; Kim et al., 2022). The genotype–phenotype gap reflects not only incomplete databases but also a representational mismatch between multidimensional resistance biology and simplified genomic features.

Context-aware models should preserve only the structural, regulatory, quantitative, background, and population information relevant to the mechanism and intended use rather than treating each determinant as an independent binary variable (**Figure 2**). A tiered, mechanism-aware framework for selecting these additional data layers and computational representations is summarized in **Table 1**. A structured comparison of the principal biological and representational sources of genotype–phenotype discordance, the limitations of conventional AST and presence–absence-based WGS analysis, and the additional data required to address them is provided in **Supplementary Table S3**.

**Figure 2:**
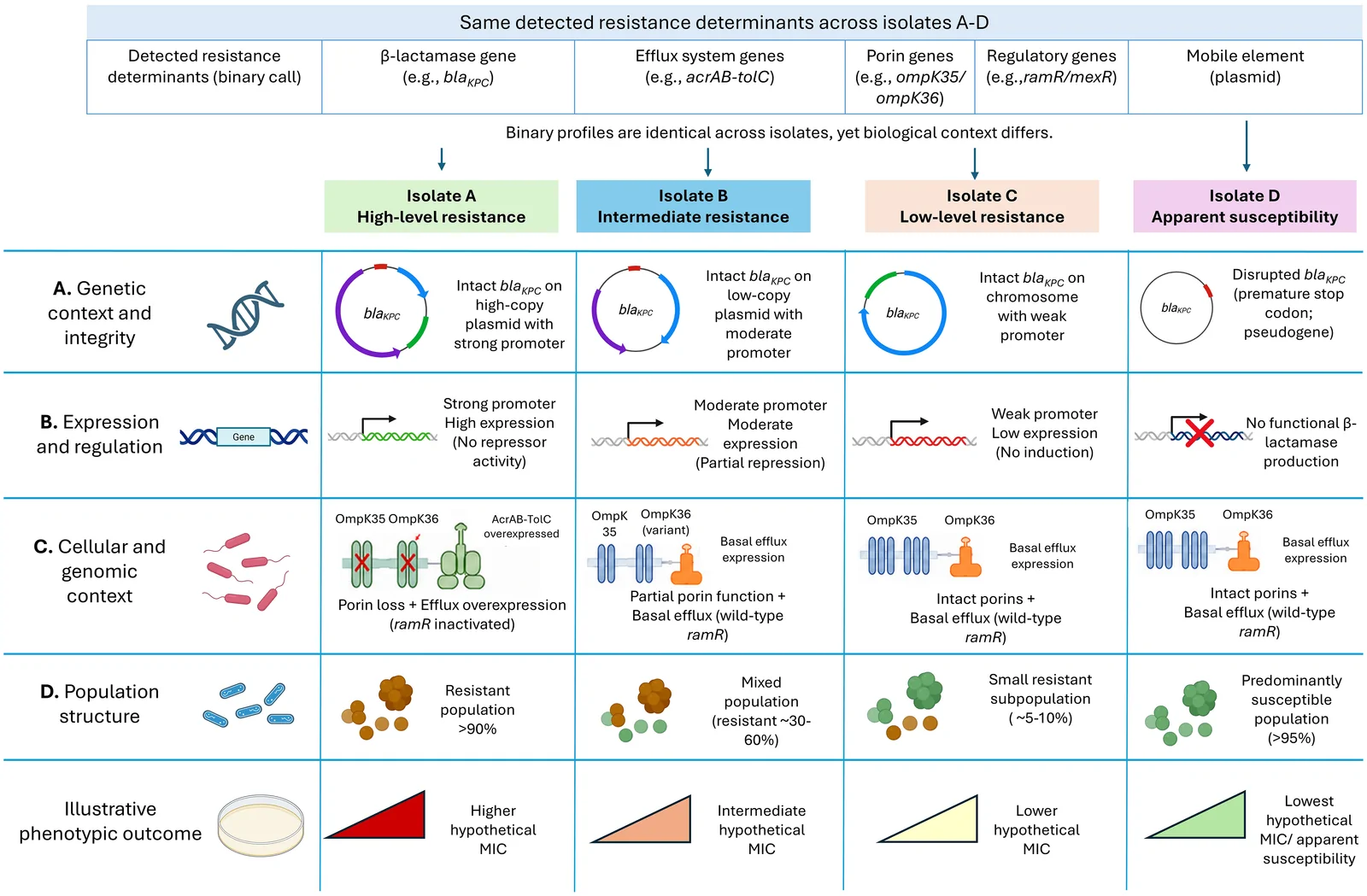
Identical binary resistome profiles can correspond to divergent antimicrobial phenotypes. **(A)** genetic context and integrity; **(B)** expression and regulation; **(C)** cellular and genomic context; and **(D)** population structure. The four hypothetical isolates share the same detected β-lactamase, efflux-system, porin, regulatory, and plasmid-associated determinants but differ in coding-sequence integrity, promoter strength, gene or plasmid copy number, efflux and porin activity, genomic background, and within-isolate population structure. These contextual differences can generate phenotypes ranging from high-level resistance to apparent susceptibility despite identical presence–absence calls. The relative MIC descriptors shown are purely illustrative and hypothetical and are used only to demonstrate conceptual divergence among isolates. They do not correspond to validated phenotypic measurements, specific antimicrobial agents, or CLSI/EUCAST clinical breakpoints and should not be interpreted as susceptibility categories. The figure highlights how binary resistome profiles collapse multidimensional biological information and why accurate phenotype prediction requires the integration of structural, regulatory, quantitative, cellular, and population-level context.

**Table 1:** Tiered, mechanism-aware implementation of context-aware AMR prediction.

Biological state/problem	Priority information	Representation	Tier/use	Limitation
Validated high-effect determinant	High-quality WGS, validated allele/variant call, quantitative MIC/AST metadata	Curated rule-based inference	Tier 1: routine core	Limited to validated organism–antimicrobial rules
Structurally disrupted determinant/pseudogene	Full-length allele, ORF integrity, truncation/active-site assessment	Sequence-integrity rules or allele-quality features	Tier 1	Functional effect of novel substitutions may remain uncertain
Functionally intact silent or attenuated determinant; efflux/porin dysregulation	Promoter/regulatory sequence; standardized expression data when needed	Regularized/tree-based or multimodal genomic-expression models	Tier 2	Expression is condition- and background-dependent
Inducible resistance (e.g., *ampC*- or *erm*-mediated resistance)	Induction phenotype; regulatory/attenuator sequence; standardized expression under inducing conditions when needed	Rule-based induction features or multimodal genomic-expression models	Tier 2	Basal testing may not reveal the inducible phenotype
Promoter strength or gene/plasmid dosage	Regulatory sequence; normalized read depth/copy number	Quantitative features with regression/classification	Tier 2	Copy-number estimates depend on sequencing depth and normalization
Genomic topology, mobile-element context, or epistasis	Gene neighborhood, plasmid/chromosome localization; long-read/hybrid sequencing when consequential	Graph/context features; graph- or attention-based candidate models	Tier 1–2	Greater computational and data requirements; incremental value requires validation
Heteroresistance	PAP; deep sequencing; minor-variant frequency; normalized read depth	Population-frequency features or mixture models	Tier 3	Detection limit is workflow-specific
Complex metagenomic One Health samples	Host linkage; long-read/target enrichment or proximity-ligation approaches (e.g., Hi-C); metatranscriptomics when needed	Host-resolved multimodal/ensemble models with uncertainty	Tier 3	Expression and ARG abundance do not establish isolate-level MIC

AMR, antimicrobial resistance; ARG, antimicrobial resistance gene; AST, antimicrobial susceptibility testing; MIC, minimum inhibitory concentration; ORF, open reading frame; PAP, population analysis profile; WGS, whole-genome sequencing. Tier 1 denotes routine core information; Tier 2, mechanism-triggered additional analysis; and Tier 3, specialized or research-level analysis. Long-read sequencing is prioritized when plasmid, structural, or neighborhood context is consequential rather than assumed to be universally required. Tiers are pragmatic and additive, not mandatory multi-omics layers.

### 4.2 Context-aware computational prediction and the limits of phenotypic labels

Prediction models require standardized phenotypic labels, yet conventional AST is context-dependent. Media, inoculum, oxygen, incubation, and predominantly planktonic growth may not reproduce induction, biofilm-associated states, or host conditions, and categorical breakpoints compress continuous MIC distributions (Humphries and Hindler, 2016; Ciofu et al., 2022; Gajic et al., 2022). Quantitative MIC values and complete AST metadata are therefore preferable for model development whenever available. Importantly, technical reproducibility of sequence detection should not be conflated with predictive validity: a genomic call may be analytically consistent yet phenotypically misleading when structural integrity, expression, dosage, genomic background, or population heterogeneity are not represented (Kim et al., 2022).

Homology-based tools should be interpreted within their intended scope: AMRFinderPlus primarily detects curated determinants/variants, whereas ResFinder 4.0 also infers phenotype for supported organism-antimicrobial combinations (Bortolaia et al., 2020; **Supplementary Table S2**). Expression, copy number, epistasis, permeability, and population heterogeneity are outside such outputs rather than failures of gene detection. Context-aware models can combine allele/ORF integrity, regulatory variants, read-depth features, gene-neighborhood/mobile-element context, and, when mechanism-specific, expression or minority-variant frequency. Rule-based systems remain appropriate for validated high-effect determinants; regularized linear or tree-based models suit sparse tabular features; graph-based or attention-based architectures are candidates for topology or regulatory sequence (Nguyen et al., 2026; Kervancı, 2026); and late-fusion ensembles can combine modalities. Missing modalities should remain explicitly unknown rather than be encoded as absence. Complex models must demonstrate incremental value, calibration, external validity, scalability, and interpretable error profiles (Nguyen et al., 2019; Kim et al., 2022; Prosperi et al., 2022).

Transcriptomics can capture efflux-pump overexpression, altered porin expression, and inducible resistance that genome-only models miss; in *Pseudomonas aeruginosa*, integrating genomic and transcriptomic features improved prediction for several agents (Khaledi et al., 2020). Nevertheless, expression profiles depend on growth conditions, antimicrobial exposure, sampling time, and RNA workflows. Routine implementation also adds cost, turnaround time, bioinformatic processing, and quality-control requirements; more broadly, clinical RNA-seq requires benchmark standards, assay optimization, and demonstration of reproducibility (Byron et al., 2016). Transcriptomics should therefore be used selectively, with standardized conditions and external validation, rather than treated as a universal replacement for genomic or phenotypic testing.

In clinical and public-health laboratories, added biological resolution must be balanced against cost, complexity, turnaround time, and quality-control burden. A pragmatic hybrid workflow is to use validated WGS-based determinant/variant prediction for routine high-concordance organism-antimicrobial pairs, then escalate to long-read/context analysis, copy-number or regulatory assessment, transcriptomics, or population-aware methods when there is unexplained genotype-phenotype discordance, an atypical MIC, a high-consequence antimicrobial, suspected heteroresistance, or a need to resolve mobile-element context. Context-aware analyses should complement rather than displace phenotypic AST when clinical consequences are high or external validation is insufficient.

### 4.3 Generalizability across species, lineages, and one health compartments

Models trained on specific species, lineages, regions, or AST procedures may achieve high internal performance by exploiting population structure rather than transferable biology (Kim et al., 2022; Prosperi et al., 2022). This limitation is especially important across One Health compartments, where animal, food, and environmental isolates remain underrepresented and may differ from clinical collections in lineage composition and genomic background (Muloi et al., 2023; Djordjevic et al., 2024). Internal cross-validation is therefore insufficient; robust evaluation requires external validation across independent lineages, geographical settings, ecological compartments, and testing frameworks, together with uncertainty estimates and assessment of performance degradation outside the training domain.

Conversely, lineage information can itself provide useful predictive context within well-characterized clonal populations when resistance determinants, accessory elements, or interacting genomic backgrounds are strongly associated with particular lineages. Lineage should therefore be represented explicitly when it improves prediction within the intended population, while lineage-aware external validation remains necessary to distinguish useful contextual signal from non-transferable population structure (Kim et al., 2022; Prosperi et al., 2022).

Evaluation design should reproduce the conditions under which a model will be applied. Random train–test partitioning may place closely related isolates in both datasets and yield optimistic performance estimates. More informative strategies include lineage-aware, geographically separated, temporally separated, and cross-compartment validation, with calibration and error rates reported for relevant subgroups. Predictive values should also be interpreted in relation to resistance prevalence because performance in an enriched development dataset may not translate directly to routine surveillance. Models encountering poorly represented species, lineages, or genomic backgrounds should communicate increased uncertainty rather than issue an unsupported categorical prediction (Kim et al., 2022; Prosperi et al., 2022).

### 4.4 Why metagenomic ARG detection does not equal phenotypic resistance

Metagenomics enables culture-independent ARG detection but often cannot assign determinants to a specific host, resolve closely related strains, or establish chromosomal, plasmid, or mobile-element context (Munk et al., 2022; Slizovskiy et al., 2022; Bloemen et al., 2025). ARG abundance also does not establish coding-sequence integrity, expression, inducibility, copy number, or population distribution. Long-read, target-enriched, or metatranscriptomic approaches may improve contextualization but still do not directly define the MIC of a particular host population.

Importantly, the co-occurrence of an ARG and a bacterial taxon within the same metagenomic sample does not demonstrate that the determinant is carried by that organism, as shown by shotgun metagenomic surveillance of farm environments, in which abundance-based genus–ARG associations could not establish physical host linkage, gene expression, or mobility context (Gomes-Gonçalves et al., 2026). Short-read assemblies may separate resistance genes from taxonomic markers, plasmid backbones, and neighboring regulatory regions, while mixed communities can generate ambiguous or incorrect linkage. Long-read sequencing and hybrid assembly can improve host and mobile-element assignment, but their resolution remains dependent on sequencing depth, community complexity, DNA quality, and the relative abundance of the organisms carrying the determinant (Slizovskiy et al., 2022; Bloemen et al., 2025).

Proximity-ligation approaches such as Hi-C provide an additional strategy for host attribution by preserving physical associations among DNA molecules within cells, allowing ARG- and plasmid-bearing sequences to be linked to candidate bacterial hosts in complex communities (Stalder et al., 2019). Such approaches can complement long-read and hybrid sequencing for resolving host–mobile-element associations, although they require specialized library preparation and bioinformatic processing and remain dependent on community complexity, sequencing depth, and analytical thresholds. Importantly, physical host linkage does not itself establish ARG expression or the antimicrobial susceptibility phenotype of the linked bacterial population.

Metatranscriptomics may provide evidence that detected ARGs are expressed, but expression alone still does not establish the susceptibility phenotype of a particular host organism. Transcript abundance may derive from multiple taxa, vary with environmental conditions, and fail to capture minority resistant populations. Accordingly, host linkage, structural context, and confirmatory phenotypic testing remain necessary when metagenomic findings are used for clinically or epidemiologically actionable interpretation. Metagenomic ARG detection should therefore be interpreted as resistance potential and, where actionable inference is required, complemented by host-resolved reconstruction, targeted isolate recovery, and confirmatory phenotypic testing (Slizovskiy et al., 2022; Bloemen et al., 2025).

## 5 Conclusions and future perspectives

The genotype–phenotype gap cannot be attributed solely to incomplete resistance-gene databases. It also reflects a representational mismatch between the multidimensional biology of antimicrobial resistance and the binary features commonly used by genomic prediction pipelines. Gene integrity, expression, dosage, regulatory topology, membrane permeability, strain background, and population heterogeneity determine whether a detected resistance determinant produces a clinically relevant phenotype. Consequently, isolates with apparently identical resistance-gene profiles may exhibit substantially different MICs, while both genomic and phenotypic methods may fail to capture transient or minority resistance states.

Future prediction frameworks should therefore move beyond determinant detection toward context-aware and quantitative representations of resistance. In this review, context-aware prediction denotes selective representation of the biological dimensions required by a given mechanism and use case, not indiscriminate multi-omics. Long-read sequencing can resolve plasmid structure, gene order, and mobile-element context; transcriptomic analysis can identify inducible expression, efflux-pump upregulation, and altered porin expression; read-depth analysis can estimate gene dosage; and population-aware sequencing can reveal low-frequency variants and amplifications. This framework is intended to refine and appropriately scope genomic prediction, not reject it: routine WGS remains highly informative for validated high-effect determinants, whereas enhanced analyses are most valuable when phenotype depends on regulation, dosage, background, structural context, or population heterogeneity.

Machine-learning approaches may integrate these heterogeneous data and identify interactions that are difficult to represent in conventional rule-based systems. However, no computational model can reliably infer biological states that are absent from its input data, and high internal performance does not establish transportability across species, lineages, geographical settings, or One Health compartments. External validation, standardized phenotypic metadata, uncertainty estimation, and confirmatory testing therefore remain essential. Genomic prediction should therefore be interpreted within validated organism-antimicrobial boundaries and as resistance potential when relevant functional context remains unresolved.
