# Supplementary material for: From genome to phenotype: advances, challenges and One Health perspectives in the genomic prediction of antimicrobial resistance
Source: Front Cell Infect Microbiol. 2026 Oct 21;16:1924354. doi: 10.3389/fcimb.2026.1924354 (PMC13732309; doi:10.3389/fcimb.2026.1924354)
Supplement: Supplementary file 1 [file Table1.docx]

Supplementary Material

# Table S1: Representative Examples of Genotype–Phenotype Discordance Associated with Resistance-Determinant Integrity, Expression, Genomic Context, and Testing Conditions

**Selection criteria:** This table is representative rather than exhaustive and was designed to span distinct taxa, antimicrobial classes, and mechanistic sources of genotype-phenotype discordance. Mechanistic statements are limited to effects directly demonstrated by the cited study; where the source documents discordance without establishing a causal mechanism, or where a restoration scenario is inferred from demonstrated biology, this is stated explicitly.

| **Bacterial species or group** | **Resistance determinant** | **Mechanistic category** | **Molecular basis of genotype–phenotype discordance** | **Observed phenotype and prediction implication** | **Potential for activation or functional restoration** | **Reference** |
| --- | --- | --- | --- | --- | --- | --- |
| Acinetobacter baumannii | *blaOXA-51-like* | Promoter-dependent expression | The endogenous carbapenemase gene is expressed at insufficient levels in the absence of an upstream insertion sequence, such as ISAba1, providing a strong promoter. | Carbapenem susceptibility or low-level resistance may occur despite gene detection; gene presence alone does not establish clinically relevant carbapenem resistance. | Activation is possible through acquisition or repositioning of an upstream insertion sequence carrying an active promoter. | (Turton et al., 2006) |
| Acinetobacter baumannii | *adeFGH* (Efflux) | Regulatory repression of efflux | Expression of the AdeFGH efflux system is repressed by AdeL and may increase following activating regulatory mutations. | Detection of efflux-pump genes does not establish overexpression or resistance to fluoroquinolones or tigecycline. | Activation is possible through mutations affecting adeL or other regulatory elements. | (Coyne et al., 2010) |
| Bacteroides fragilis | *cfiA* | Promoter-dependent expression | The metallo-β-lactamase gene may lack an effective native promoter and require an upstream insertion sequence for clinically relevant expression. | Carbapenem susceptibility may occur despite the presence of *cfiA*. | Activation is possible following insertion-sequence acquisition or another promoter-generating event. | (Podglajen et al., 1992) |
| Enterobacterales | *blaACC-1* | Altered substrate spectrum and phenotypic-screening limitation | ACC-1 differs from many AmpC β-lactamases because it has limited activity against cephamycins such as cefoxitin. | Cefoxitin susceptibility may prevent recognition by conventional AmpC screening. Conversely, gene presence should not automatically be interpreted as cefoxitin resistance. | Not a silencing or reactivation phenomenon; interpretation requires enzyme- and drug-specific knowledge. | (Jacoby, 2009) |
| Enterobacterales | *blaNDM-1* | Testing-medium and cofactor dependence | NDM enzymatic activity depends on zinc availability, which varies among Mueller–Hinton broth formulations. | Zinc-depleted media may produce falsely low meropenem MICs despite the presence of a functional metallo-β-lactamase. | No genetic activation is required; the measured phenotype varies with cofactor availability and testing conditions. | (Bilinskaya et al., 2020) |
| Enterobacterales | *mcr-9* | Inducible expression | Expression may depend on activation by the QseBC two-component regulatory system and remain low under non-inducing conditions. | Isolates may test susceptible to colistin despite carrying *mcr-9*. | Induction is possible through the appropriate regulatory and environmental signals when a functional regulatory system is present. | (Kieffer et al., 2019) |
| Enterococcus faecium | *vanA* operon | Structural disruption or pseudogenization | Major deletions within Tn1546 or the *vanA* operon disrupt the resistance pathway. | The isolate may remain susceptible to vancomycin and teicoplanin despite detection of residual *vanA*-associated sequences. Homology-based annotation may therefore overcall resistance. | Not expected to reactivate through antimicrobial exposure alone; restoration would require recombination, reversion, or acquisition of an intact operon. | (Gagnon et al., 2011) |
| Enterococcus spp. | *optrA*, *poxtA* | Breakpoint-dependent carriage and selection-dependent resistance | In *Enterococcus* isolates with a linezolid MIC of 4 mg/L (susceptible under the EUCAST breakpoint used in the study), resistance genes and/or G2576T mutations were detected in 40%. The cited study did not establish uniformly low basal expression as the cause of susceptibility. | Resistance determinants can coexist with a susceptible category at the breakpoint; during in vitro linezolid selection, some determinant-positive isolates increased above the clinical breakpoint. | Selection-dependent MIC increases were demonstrated for a subset of isolates; the mechanism and probability are background-dependent and should not be inferred from gene presence alone. | (Bender et al., 2024) |
| Escherichia coli | *blaOXA-2, aadA1, sul1, tetA* | Transcriptional silencing | Chromosomal context or local transcriptional control may override otherwise functional promoter and coding sequences. | Susceptibility may occur despite the presence of structurally intact resistance genes. | Activation may be possible through changes in local regulation, genomic context, or selective conditions, depending on the determinant. | (Enne et al., 2006) |
| Escherichia coli | *mcr-1* | Promoter-dependent low expression | Among 15 *mcr-1*-positive clinical Enterobacteriaceae, one *E. coli* isolate (EC09) remained colistin susceptible (MIC 0.5 mg/L) and showed the lowest *mcr-1* transcription and promoter activity. | Gene carriage can coexist with colistin susceptibility when expression is insufficient; the cited study directly associated lower promoter activity/expression with the susceptible isolate. | Higher expression can increase colistin MIC, but activation of a given isolate should not be assumed without direct regulatory evidence. | (Lu et al., 2021) |
| Escherichia coli | *strA, strB, aadA* | Breakpoint-related genotype–phenotype discordance | Structurally intact determinants may produce increases in MIC that remain below the selected clinical breakpoint. | Isolates may be categorized as susceptible to streptomycin despite measurable genotypic resistance potential. | Not an activation phenomenon; the discordance results principally from phenotype interpretation and breakpoint selection. | (Tyson et al., 2015) |
| Gram-negative bacteria | Integron gene cassettes | Topological attenuation / promoter-distance effect | Experimental constructs showed that resistance was highest when a cassette was first in the array and decreased when the same cassette was positioned downstream from the common promoter. | The same cassette can produce different expression levels and resistance magnitudes depending on cassette order and promoter distance. | Moving a cassette promoter-proximal would be expected to increase expression based on the demonstrated position effect; that restoration scenario was not directly tested in the cited study. | (Collis and Hall, 1995) |
| Klebsiella pneumoniae | *blaCTX-M* | Upstream promoter / insertion-sequence context | ISEcp1 can provide promoter activity for *blaCTX-M* and participate in its mobilization; the expression potential therefore depends on preservation and orientation of the upstream activating context. | Detection of the *blaCTX-M* coding sequence alone may not fully represent expression when the upstream context is altered or separated. | Restoration would require an active upstream promoter context; the cited study demonstrates promoter contribution rather than a universal reactivation pathway. | (Poirel et al., 2003) |
| Klebsiella pneumoniae | *qnrB* | Low-level resistance below categorical breakpoint | *qnrB* can produce a modest increase in fluoroquinolone MIC that may remain below a clinical resistance breakpoint. | Apparent ciprofloxacin susceptibility may coexist with a transferable quinolone-resistance determinant. | Not necessarily a silencing phenomenon; phenotype depends on determinant effect size and additional background mechanisms. | (Jacoby et al., 2006) |
| Klebsiella pneumoniae | *blaKPC-71* | Allele-specific functional alteration | An in-frame nucleotide insertion produces an amino-acid duplication near the active site, modifying the enzyme’s substrate profile. | Carbapenem MICs may decrease while resistance to ceftazidime–avibactam is maintained or increased. Gene-family identification alone cannot predict the resulting phenotype. | Not conditionally silent. Restoration of the ancestral phenotype would require reversion, compensatory evolution, or acquisition of another allele. | (Yang et al., 2025) |
| Mammaliicoccus sciuri (formerly Staphylococcus sciuri) | *mecA*  homologue | Transcriptional activation of a native homologue | Most isolates can be β-lactam susceptible despite ubiquitous carriage of the native *mecA* homologue. In resistant isolates, high transcription was linked to an upstream IS256 insertion or promoter substitutions. | Gene carriage alone does not establish methicillin resistance; experimentally observed promoter activation was associated with increased transcription and resistance. | Transcriptional activation through promoter-region changes or insertion-sequence acquisition was directly demonstrated. | (Couto et al., 2003) |
| Salmonella enterica serovar Enteritidis | *aac(6')-Iy* | Cryptic determinant requiring genomic rearrangement | The chromosomal gene remains transcriptionally inactive until a large deletion generates a transcriptional fusion with an active promoter. | Aminoglycoside susceptibility may occur despite ubiquitous carriage of the gene. | Activation is possible, but requires a relatively rare genomic rearrangement rather than ordinary environmental induction. | (Magnet et al., 1999) |
| Staphylococcus aureus | *mecA* | Background-dependent expression and adaptive transition | Oxacillin-susceptible *mecA*-positive *S. aureus* can acquire increased β-lactam resistance through a combination of *mecA* upregulation and auxiliary/background mutations; experimental *mecA* overexpression increased oxacillin MIC. | *mecA* detection alone can overcall the current phenotype in oxacillin-susceptible *mecA*-positive isolates, although the genotype can retain potential to evolve resistance under selection. | Increased resistance can emerge through *mecA* upregulation plus background adaptation; the cited evidence does not support reducing this phenomenon to MecI/MecR1 repression alone. | (Boonsiri et al., 2020) |
| Streptococcus pneumoniae | *erm(B)* | Inducible expression | Expression depends on translational attenuation and ribosomal stalling in the presence of an appropriate macrolide inducer. | Clindamycin susceptibility may be observed when testing does not induce MLSB resistance. | Readily inducible when the attenuator and coding sequence remain functional and the appropriate inducer is present. | (Leclercq, 2002) |

**Abbreviations:** AMR, antimicrobial resistance; MIC, minimum inhibitory concentration; MLSB, macrolide-lincosamide-streptogramin B. Gene symbols should be interpreted as determinants detected at the sequence level unless expression or function was directly measured. “Activation” is used only for structurally intact determinants whose expression or resistance phenotype can increase through induction, promoter/regulatory change, or genomic rearrangement. Structurally disrupted genes or pseudogenes are not expected to regain function through exposure alone and would require reversion, recombination, compensatory evolution, or acquisition of an intact allele.

**Table S2: Representative Computational Tools and Feature Representations Used in AMR Genotype-to-Phenotype Analysis**

**Selection criteria.** Table S2 is illustrative rather than a benchmark or ranking. Tools were selected to represent commonly used determinant-detection, rule-based phenotype-inference, k-mer machine-learning, pathogen-specific, and multimodal approaches. Listed limitations describe biological information outside each tool’s intended input/design and should not be interpreted as defects in software quality.

| **Tool /platform** | **Main approach** | **Input data** | **Main output** | **Relevance for genotype-to-phenotype prediction** | **Biological Information not represented** | **Main limitation/scope** | **References** |
| --- | --- | --- | --- | --- | --- | --- | --- |
| AMRFinderPlus | Curated detection of AMR genes, resistance-associated point mutations and selected stress/virulence genes | Protein annotations and/or assembled nucleotide sequences | AMR genes, point mutations and related genetic elements | Highly standardized and widely used for surveillance annotation; useful for interpreting known resistance determinants | Expression state, copy number, population heterogeneity | Designed primarily for determinant/variant detection rather than direct MIC modelling; phenotype inference requires organism/drug-specific interpretation. | (Feldgarden et al., 2021) |
| ARIBA | Local assembly of short reads against reference AMR genes and mutations | Paired-end short reads | AMR-associated genes, SNPs and sequence quality details | Useful because it works directly from reads and can detect variation relative to reference sequences | Regulatory state and phenotype magnitude | Reference-driven; performance depends on database completeness and known markers. | (Hunt et al., 2017) |
| CARD / RGI | Homology- and SNP-model-based resistome prediction using the CARD ontology | Genomes, assemblies, proteins, metagenomic contigs or reads | Predicted resistome, AMR gene families, resistance mechanisms and drug classes | Strong for systematic resistome annotation and mechanism-level interpretation | Functional activity of detected determinants | Detects resistance potential; gene detection alone does not establish phenotypic resistance. | (Alcock et al., 2023) |
| Kover / k-mer ML models | Interpretable machine learning using k-mers | Whole-genome sequence data converted into k-mer features | Phenotype classifiers and genotype–phenotype associations | Captures markers beyond curated ARGs and may identify novel associations | Causal interpretation and population-state information | May learn lineage or dataset structure rather than transferable causal mechanisms. | (Drouin et al., 2016) |
| Mykrobe | Genotype-based resistance prediction using curated mutation/gene catalogues | Raw sequencing reads | Species identification and resistance predictions, especially for *M. tuberculosis* and *S. aureus* | More explicitly phenotype-oriented than simple ARG scanners; designed to generate resistance prediction reports | Expression state, gene dosage, population heterogeneity, and mechanisms outside the curated catalogue. | Species- and catalogue-specific; not directly transferable beyond validated organism-antimicrobial combinations. | (Bradley et al., 2015) |
| ResFinder / PointFinder | Rule-based detection of acquired ARGs and resistance-associated chromosomal mutations | Assembled genomes or sequence data | Detected resistance genes/mutations and predicted resistance phenotypes for supported species–drug combinations | One example of curated genotype-to-phenotype inference; ResFinder 4.0 was specifically developed for phenotype prediction from genotypes | Inducible, regulatory, polygenic and background-dependent resistance. | Limited to known mechanisms and supported organism-antimicrobial combinations; boundaries reflect intended scope rather than gene-detection failure. | (Bortolaia et al., 2020) |
| seq2geno2pheno / Model-T | Machine learning integrating genomic and transcriptomic features | WGS, RNA-seq and paired susceptibility data | Susceptibility classifiers and predictive molecular markers | Captures expression-dependent mechanisms, including efflux and porin regulation, that are missed by genome-only models. | Expression outside the tested conditions and minority resistant subpopulations | Species- and condition-specific; transcriptomic standardization limits routine transferability. | (Khaledi et al., 2020) |
| staramr | BLAST-based screening against ResFinder, PointFinder and PlasmidFinder | Bacterial genome assemblies | ARGs, point mutations, plasmid replicons and summary reports | Practical surveillance integration of resistance determinants, resistance-associated mutations, and plasmid replicon calls. | Expression and functional viability | Annotation/inference tool dependent on underlying databases; detection of an ARG and a plasmid replicon does not by itself establish their physical linkage, and the tool does not resolve expression or functional activity. | (PHAC-NML, n.d.) |
| TB-Profiler | Variant-based prediction using curated *M. tuberculosis* resistance markers | FASTQ reads, alignments or variants | *M. tuberculosis* lineage and drug-resistance prediction | Strong example of pathogen-specific genomic DST, with curated resistance databases and large validation datasets | Expression state and resistance mechanisms outside the curated marker catalogue | Restricted to *M. tuberculosis* and validated drug-resistance rules. | (Phelan et al., 2019) |

**Table S3:** **Biological and Representational Sources of Genotype–Phenotype Discordance and the Data Required to Address Them**

| **Source of Discordance** | **Phenotypic consequence or limitation of conventional AST** | **Why standard WGS/presence–absence may fail** | **Additional information or analytical approach** |
| --- | --- | --- | --- |
| **Efflux-pump overexpression** | Expression may vary with growth conditions and antimicrobial exposure. | Presence of *acrAB-tolC, mexAB-oprM*, or related loci does not establish overexpression | Regulatory-variant analysis; transcriptomics; efflux phenotyping. |
| **Functionally intact but attenuated or conditionally expressed ARGs** | The determinant may remain uninduced under routine testing conditions | Gene presence does not establish transcriptional or translational activity | Transcriptomics under standardized conditions; promoter and regulatory-region analysis; induction testing |
| **Genomic topology and epistasis** | The same determinant may produce different phenotypes in different backgrounds | Binary matrices discard gene order, promoter proximity, mobile-element context, and interacting mechanisms | Long-read sequencing and context-aware models; external validation remains required |
| **Heteroresistance** | Bulk AST may miss minor resistant subpopulations | Consensus sequencing may mask low-frequency variants or transient amplifications | Population analysis profile (PAP); targeted deep sequencing; read-depth and minor-variant analysis |
| **Inducible resistance** | Basal testing may not induce *ampC* or *erm* expression | Gene presence does not distinguish basal, inducible, derepressed, or constitutive states | Induction assays; D-test; regulatory-region analysis; transcriptomics |
| **Porin loss or alteration** | Reduced influx may increase MIC only in combination with other mechanisms. | Binary ARG detection does not represent OmpK35/OmpK36 or OprD expression and functionality. | Coding-variant, promoter, and expression analysis. |
| **Promoter strength and gene dosage** | Different expression levels may produce different MICs | Presence–absence matrices do not represent promoter variants or copy number | Promoter-sequence analysis; normalized read depth; plasmid copy-number estimation |
| **Structurally disrupted ARGs or pseudogenes** | The isolate may be genuinely susceptible; discordance arises from genotype-based overcalling rather than from failure of phenotypic testing. | Homology-based pipelines may report a disrupted or truncated gene as present | Full-length allele analysis; open-reading-frame integrity; truncation and active-site assessment. |
| **Testing-medium and cofactor dependence** | Measured MICs may vary according to medium composition, ion availability, inoculum, or other assay conditions. | Genome sequence does not represent the biochemical conditions under which the phenotype was measured. | Standardized media, condition-specific susceptibility testing, and explicit recording of assay metadata. |

**Figure S1:** Broader framework for integrated One Health genomic AMR surveillance and prediction. This five-step architecture illustrates the broader implementation context for genomic antimicrobial resistance surveillance: (1) integrated sampling across human, animal, food, and environmental sectors; (2) selective incorporation of genomic, transcriptomic, and phenotypic inputs according to the resistance mechanism and intended use; (3) data and metadata harmonization; (4) predictive modelling with external validation and uncertainty assessment; and (5) translation into clinical, epidemiological, and public-health decision support. The figure is a conceptual framework rather than a validated universal workflow.

**References:**

Alcock, B. P., Huynh, W., Chalil, R., Smith, K. W., Raphenya, A. R., Wlodarski, M. A., et al. (2023). CARD 2023: expanded curation, support for machine learning, and resistome prediction at the Comprehensive Antibiotic Resistance Database. *Nucleic Acids Res.* 51, D690–D699. doi: 10.1093/nar/gkac920

Bender, J. K., Fleige, C., Funk, F., Moretó-Castellsagué, C., Fischer, M. A., and Werner, G. (2024). Linezolid resistance genes and mutations among linezolid-susceptible *Enterococcus* spp.—A loose cannon? *Antibiotics* 13, 101. doi: 10.3390/antibiotics13010101

Bilinskaya, A., Buckheit, D. J., Gnoinski, M., Asempa, T. E., and Nicolau, D. P. (2020). Variability in zinc concentration among Mueller-Hinton broth brands: impact on antimicrobial susceptibility testing of metallo-β-lactamase-producing *Enterobacteriaceae*. *J. Clin. Microbiol.* 58, e02019-20. doi: 10.1128/jcm.02019-20

Boonsiri, T., Watanabe, S., Tan, X. E., Thitiananpakorn, K., Narimatsu, R., Sasaki, K., et al. (2020). Identification and characterization of mutations responsible for the β-lactam resistance in oxacillin-susceptible *mecA*-positive *Staphylococcus aureus*. *Sci. Rep.* 10, 16907. doi: 10.1038/s41598-020-73796-5

Bortolaia, V., Kaas, R. S., Ruppe, E., Roberts, M. C., Schwarz, S., Cattoir, V., et al. (2020). ResFinder 4.0 for predictions of phenotypes from genotypes. *J. Antimicrob. Chemother.* 75, 3491–3500. doi: 10.1093/jac/dkaa345

Bradley, P., Gordon, N. C., Walker, T. M., Dunn, L., Heys, S., Huang, B., et al. (2015). Rapid antibiotic-resistance predictions from genome sequence data for *Staphylococcus aureus* and *Mycobacterium tuberculosis*. *Nat. Commun.* 6, 10063. doi: 10.1038/ncomms10063

Collis, C. M., and Hall, R. M. (1995). Expression of antibiotic resistance genes in the integrated cassettes of integrons. *Antimicrob. Agents Chemother.* 39, 155–162. doi: 10.1128/aac.39.1.155

Couto, I., Wu, S. W., Tomasz, A., and De Lencastre, H. (2003). Development of methicillin resistance in clinical isolates of *Staphylococcus sciuri* by transcriptional activation of the *mecA* homologue native to *Staphylococcus sciuri*. *J. Bacteriol.* 185, 645–653. doi: 10.1128/jb.185.2.645-653.2003

Coyne, S., Rosenfeld, N., Lambert, T., Courvalin, P., and Périchon, B. (2010). Overexpression of resistance-nodulation-cell division pump *adeFGH* confers multidrug resistance in *Acinetobacter baumannii*. *Antimicrob. Agents Chemother.* 54, 4389–4393. doi: 10.1128/aac.00155-10

Drouin, A., Giguère, S., Déraspe, M., Marchand, M., Tyers, M., Loo, V. G., et al. (2016). Predictive computational phenotyping and biomarker discovery using reference-free genome comparisons. *BMC Genomics* 17, 268. doi: 10.1186/s12864-016-2889-6

Enne, V. I., Delsol, A. A., Roe, J. M., and Bennett, P. M. (2006). Evidence of antibiotic resistance gene silencing in *Escherichia coli*. *Antimicrob. Agents Chemother.* 50, 3003–3010. doi: 10.1128/aac.00137-06

Feldgarden, M., Brover, V., Gonzalez-Escalona, N., Frye, J. G., Haendiges, J., Haft, D. H., et al. (2021). AMRFinderPlus and the Reference Gene Catalog facilitate examination of the genomic links among antimicrobial resistance, stress response, and virulence. *Sci. Rep.* 11, 12728. doi: 10.1038/s41598-021-91456-0

Gagnon, S., Lévesque, S., Lefebvre, B., Bourgault, A. M., Labbé, A. C., and Roger, M. (2011). *vanA*-containing *Enterococcus faecium* susceptible to vancomycin and teicoplanin because of major nucleotide deletions in *Tn1546*. *J. Antimicrob. Chemother.* 66, 2758–2762. doi: 10.1093/jac/dkr379

Hunt, M., Mather, A. E., Sánchez-Busó, L., Page, A. J., Parkhill, J., Keane, J. A., et al. (2017). ARIBA: rapid antimicrobial resistance genotyping directly from sequencing reads. *Microb. Genom.* 3, e000131. doi: 10.1099/mgen.0.000131

Jacoby, G. A. (2009). *AmpC* β-lactamases. *Clin. Microbiol. Rev.* 22, 161–182. doi: 10.1128/cmr.00036-08

Jacoby, G. A., Walsh, K. E., Mills, D. M., Walker, V. J., Oh, H., Robicsek, A., et al. (2006). *qnrB*, another plasmid-mediated gene for quinolone resistance. *Antimicrob. Agents Chemother.* 50, 1178–1182. doi: 10.1128/aac.50.4.1178-1182.2006

Khaledi, A., Weimann, A., Schniederjans, M., Asgari, E., Kuo, T., Oliver, A., et al. (2020). Predicting antimicrobial resistance in *Pseudomonas aeruginosa* with machine learning-enabled molecular diagnostics. *EMBO Mol. Med.* 12, e10264. doi: 10.15252/emmm.201910264

Kieffer, N., Royer, G., Decousser, J. W., Bourrel, A. S., Palmieri, M., De La Rosa, J. M. O., et al. (2019). *mcr-9*, an inducible gene encoding an acquired phosphoethanolamine transferase in *Escherichia coli*, and its origin. *Antimicrob. Agents Chemother.* 63, e00965-19. doi: 10.1128/aac.00965-19

Leclercq, R. (2002). Mechanisms of resistance to macrolides and lincosamides: nature of the resistance elements and their clinical implications. *Clin. Infect. Dis.* 34, 482–492. doi: 10.1086/324626

Lu, S., Li, D., Wang, L., Bi, Y., Wang, M., and Yang, F. (2021). Promoter variations associated with expression of *mcr-1* gene and level of colistin resistance. *Int. J. Antimicrob. Agents* 58, 106371. doi: 10.1016/j.ijantimicag.2021.106371

Magnet, S., Courvalin, P., and Lambert, T. (1999). Activation of the cryptic *aac(6′)-Iy* aminoglycoside resistance gene of *Salmonella* by a chromosomal deletion generating a transcriptional fusion. *J. Bacteriol.* 181, 6650–6655. doi: 10.1128/jb.181.21.6650-6655.1999

PHAC-NML (n.d.). *staramr: Scans genome contigs against the ResFinder, PlasmidFinder, and PointFinder databases*. Available at: https://github.com/phac-nml/staramr (Accessed June 11, 2026).

Phelan, J. E., O’Sullivan, D. M., Machado, D., Ramos, J., Oppong, Y. E. A., Campino, S., et al. (2019). Integrating informatics tools and portable sequencing technology for rapid detection of resistance to anti-tuberculous drugs. *Genome Med.* 11, 41. doi: 10.1186/s13073-019-0650-x

Podglajen, I., Breuil, J., Bordon, F., Gutmann, L., and Collatz, E. (1992). A silent carbapenemase gene in strains of *Bacteroides fragilis* can be expressed after a one-step mutation. *FEMS Microbiol. Lett.* 91, 21–29. doi: 10.1016/0378-1097(92)90557-5

Poirel, L., Decousser, J. W., and Nordmann, P. (2003). Insertion sequence *ISEcp1B* is involved in expression and mobilization of a *bla*CTX-M beta-lactamase gene. *Antimicrob. Agents Chemother.* 47, 2938–2945. doi: 10.1128/aac.47.9.2938-2945.2003

Turton, J. F., Ward, M. E., Woodford, N., Kaufmann, M. E., Pike, R., Livermore, D. M., et al. (2006). The role of *ISAba1* in expression of *OXA* carbapenemase genes in *Acinetobacter baumannii*. *FEMS Microbiol. Lett.* 258, 72–77. doi: 10.1111/j.1574-6968.2006.00195.x

Tyson, G. H., McDermott, P. F., Li, C., Chen, Y., Tadesse, D. A., Mukherjee, S., et al. (2015). WGS accurately predicts antimicrobial resistance in *Escherichia coli*. *J. Antimicrob. Chemother.* 70, 2763–2769. doi: 10.1093/jac/dkv186

Yang, Z., Xu, S., Xiao, Z., Xu, D., and Tao, Q. (2025). Reversible evolution of ceftazidime-avibactam resistance driven by *bla*KPC-71 and aerobactin loss in ST11-KL64 hypervirulent *Klebsiella pneumoniae*. *Infect. Drug Resist.* 18, 6407–6420. doi: 10.2147/idr.s565339
